# Post mortem magnetic resonance imaging in the fetus, infant and child: A comparative study with conventional autopsy (MaRIAS Protocol)

**DOI:** 10.1186/1471-2431-11-120

**Published:** 2011-12-22

**Authors:** Sudhin Thayyil, Neil J Sebire, Lyn S Chitty, Angie Wade, Oystein Olsen, Roxana S Gunny, Amaka Offiah, Dawn E Saunders, Catherine M Owens, WK 'Kling' Chong, Nicola J Robertson, Andrew M Taylor

**Affiliations:** 1 Institute for Women's Health, University College London, London, UK; 2 Institute of Child Health, University College London, London, UK; 3 Centre for Paediatric Epidemiology and Biostatistics, Institute of Child Health, University College London, London, UK; 4 Great Ormond Street Hospital for Children, London, UK; 5 Sheffield Children's Hospital, Sheffield; 6 Institute of Cardiovascular Science, University College London, London, UK

**Keywords:** Autopsy, post mortem magnetic resonance imaging, stillbirth, sudden infant death, diagnostic study, minimally invasive autopsy

## Abstract

**Background:**

Minimally invasive autopsy by post mortem magnetic resonance (MR) imaging has been suggested as an alternative for conventional autopsy in view of the declining consented autopsy rates. However, large prospective studies rigorously evaluating the accuracy of such an approach are lacking. We intend to compare the accuracy of a minimally invasive autopsy approach using post mortem MR imaging with that of conventional autopsy in fetuses, newborns and children for detection of the major pathological abnormalities and/or determination of the cause of death.

**Methods/Design:**

We recruited 400 consecutive fetuses, newborns and children referred for conventional autopsy to one of the two participating hospitals over a three-year period. We acquired whole body post mortem MR imaging using a 1.5 T MR scanner (Avanto, Siemens Medical Solutions, Enlargen, Germany) prior to autopsy. The total scan time varied between 90 to 120 minutes. Each MR image was reported by a team of four specialist radiologists (paediatric neuroradiology, paediatric cardiology, paediatric chest & abdominal imaging and musculoskeletal imaging), blinded to the autopsy data. Conventional autopsy was performed according to the guidelines set down by the Royal College of Pathologists (UK) by experienced paediatric or perinatal pathologists, blinded to the MR data. The MR and autopsy data were recorded using predefined categorical variables by an independent person.

**Discussion:**

Using conventional post mortem as the gold standard comparator, the MR images will be assessed for accuracy of the anatomical morphology, associated lesions, clinical usefulness of information and determination of the cause of death. The sensitivities, specificities and predictive values of post mortem MR alone and MR imaging along with other minimally invasive post mortem investigations will be presented for the final diagnosis, broad diagnostic categories and for specific diagnosis of each system.

**Clinical Trial Registration:**

NCT01417962

**NIHR Portfolio Number: **6794

## Background

For over 500 years a post mortem examination has been used to establish cause of death. This procedure provides valuable information on pathological processes - one of the key foundations of medical education. Perinatal and neonatal post mortem examination has a particularly valuable role; this was formally recognised some 15 years ago when the Royal College of Obstetricians and Gynaecologists (RCOG) and Royal College of Pathologists recommended that a perinatal post mortem examination rate of less than 75% was unacceptable and that the ideal was 100% [1,2]. Autopsy rates have steadily declined over the years since this document was published [2-5]. This decline has been accelerated by adverse publicity surrounding alleged organ retention without formal parental consent in the Bristol Royal Infirmary Inquiry [6] and the Royal Liverpool Children's Inquiry [7]. Neonatal post mortem examination consent rate was less than 20% in England and Wales in the most recent report of the Confidential Enquiry into Stillbirths and Deaths in Infancy (CESDI) [8].

The loss of a fetus, baby or child is devastating to parents. As well as coping with their loss, parents often want to know why their child died, and if there is an increased risk for existing children or for future pregnancies. A post mortem examination may provide this information. In 14-46% of perinatal and infant post mortem examinations, additional clinically significant information is found beyond that known prior to the examination, which would affect counselling or recurrence risks [3,4,9,10]. The findings may confirm or refute clinical diagnoses made during life. Many studies report significant disagreement between the pre-morbid diagnosis and post mortem examination in at least 10% of cases. This impacts both upon recurrence risks and the approach to prenatal diagnosis in future pregnancies [3,11]. Post mortem examination thus has a valuable place in confirming or refuting pre-morbid diagnoses, making further diagnoses and identifying genetic and obstetric factors of relevance to the management of future pregnancies, allowing appropriate counselling of families who can then make informed, reproductive choices. The post mortem examination will also provide useful information for clinicians, helping them to understand the causes and effects of diseases as well as the effectiveness and complications of treatment. In addition, the post mortem examination can play a crucial role in research and so advance the progress of fetal and paediatric medicine.

Should a post mortem examination be performed, recent alterations to the post mortem examination procedure and consent process may reduce the amount of information available, especially for central nervous system abnormalities [12-14]. Until recently, the usual practice was to remove and fix the brain before dissection, a process that could take up to 3 weeks. Parents now frequently request that all organs are replaced before burial. As adequate fixation is difficult within this time, the brain has to be examined following a suboptimal period of fixation, which can make interpretation of the developing brain difficult. Delay between intrauterine death and delivery, leading to maceration of the fetus, makes brain examination more difficult for the pathologist. The RCOG guidelines state that any pregnancy terminated after 22 weeks gestation should be accompanied by fetocide to ensure that the fetus is not born alive. This procedure is usually accompanied by the administration of mifepristone (a cervical ripening drug), which has its optimum efficacy in shortening the time between induction and delivery after 48 hours [15]. This effectively means that most delivered fetuses undergoing termination of pregnancy after 22 weeks gestation will have been dead for at least 48 hours, rendering post mortem examination of the brain difficult.

In addition to the difficulties of acquiring consent to perform conventional autopsy, and sufficient time to perform optimal histological preparation, various religious communities find conventional autopsy unacceptable [1]. Provision of a less invasive, accurate and widely available method of post mortem assessment has been advocated [1] and would enable access to post mortem information for the first time for many in these communities.

In summary, a less invasive method of accurately assessing detailed anatomical and pathological changes in all body systems after death would be of great value. Information for diagnosis and clinical audit can be obtained as well as creating a permanent electronic record of findings, whilst allaying parental concern with regard to organ retention or conventional invasive post mortems.

Though conventional radiology to assess the chest and bones has been used for some time in post mortem examination (for example, a skeletal survey is performed on all paediatric cases referred to the coroner and in all perinatal cases), MR imaging would be well suited as a non-invasive imaging modality for post mortem assessment. Standard imaging protocols could be performed in any hospital equipped with an MR scanner and the images sent to a centre of expertise for reporting. MR imaging would potentially overcome some of the weaknesses of conventional autopsy, providing a complete multisystem analysis that is non-invasive.

MR imaging of the excised brain [16], spine [17] and heart [18] has been successfully performed. However, although an initial feasibility study of whole-body post mortem MR imaging was reported in 1996 [19], its use in clinical practice has remained controversial. Several small studies of whole-body post mortem MR in fetuses have been reported [20-22]. In all fetal studies, imaging of the central nervous system (CNS) proved the most accurate, whilst body imaging, in particular imaging of the heart proved more problematic. A recent study, focussed on the diagnosis of CNS abnormalities in fetuses and stillbirths, reported a sensitivity of 100% and specificity 92% for MR compared with conventional post mortem examination [23]. Other studies have confirmed the accuracy of CNS fetal post mortem MR [24,25]. Imaging of the other body systems has been less well documented. Our own recent experience is that accurate post mortem body MR image acquisition is possible with modern MR imaging sequences (unpublished data), but post mortem MR imaging of the heart is less accurate [26-28].

Furthermore, MR imaging has grown in clinical importance in the living fetus and newborn infant [29], especially for brain anomalies. There is now extensive literature describing the normal MR imaging appearance of the *in utero *fetal brain from around 17 weeks gestation [30] and the *ex-utero *preterm infant brain from around 25 weeks gestation [31].

The decline in parental consent for autopsy, and technical limitations of conventional autopsy to define some nervous system abnormalities, together with a reduction in number of skilled perinatal pathologists and morphologists, has lead to a need to seek alternative less invasive methods for post mortem examination of the fetus, neonate and child. In 2001, the UK Chief Medical Officer recommended that modern imaging methods should be evaluated [32]. Since then several reports on forensic aspects of post mortem imaging have been published, however, these studies are limited to post mortem imaging in adults, primarily using computerised tomography (CT) and many studies were of poor quality [33].

Our previously published systematic review on post mortem MR imaging in fetuses, newborns, children and adults demonstrates that there is insufficient evidence to recommend the use of post mortem MR imaging as an alternative for conventional autopsy [34]. Most comparative studies to date have been small and/or have compared single systems such as the brain and did not have adequate blinding of radiologists and pathologists due to their retrospective nature. In particular, none have (a) systematically examined all the body systems in a large series of fetal, neonatal and childhood deaths (b) assessed the MR appearance of death-induced artefacts or the effect that death and maceration may have on the MR image (c) have assessed the possible disadvantages or advantages of a minimally invasive post mortem examination in combination with MR imaging.

Thus, over a decade after the first description of post mortem MR imaging, we still lack the evidence for routine implementation. Here we describe a large, prospective, blinded, comparative study to evaluate MR as an alternative to conventional invasive autopsy in fetuses, newborns and children.

### Hypothesis

MR imaging can provide an accurate, detailed, three-dimensional post mortem record of structural abnormalities and the disease processes of the whole body in the fetus, neonate and child, with similar diagnostic information to a conventional autopsy.

### Primary objective

To compare the accuracy of whole body post mortem MR imaging for detecting the cause of death and/or major pathological lesions with that of conventional autopsy in fetuses, newborns and children.

### Secondary objectives

To compare ante mortem imaging assessment (ultrasound and MR) of fetuses with post mortem MR and CT images.

To compare ante mortem diagnosis, including imaging data, in neonates, infants and children with post mortem MR images.

## Methods/Design

The study has been ongoing at two hospitals: Great Ormond Street Hospital for Children NHS Trust (GOSH) and University College Hospital NHS Foundation Trust (UCH), since March 2007. These hospitals are associated with a single academic institution - University College London (UCL). All recruited cases underwent post mortem MR imaging at 1.5 T (Avanto, Siemens Medical Solutions, Enlargen, Germany) as well as a conventional autopsy. CT imaging was also performed in cases with suspected traumatic injury or skeletal dysplasia.

In line with the CESDI recommendations, post mortem examination was offered in all cases of perinatal death and consent sought by the appropriately trained staff (consultant, experienced nurse, or experienced midwife). For consented autopsies, the standard National Health Service (NHS) consent form (produced by Department of Health) that includes consenting for the use of post mortem imaging for research was used (consent form and patient information sheet given in appendix 1 and 2) [35,36].

In Her Majesty's (HM) Coroner's cases no parental consent for autopsy was required and so, once the body was received by the GOSH mortuary, a member of the research team contacted the HM coroner's office for permission for a bereavement nurse to approach the parents by telephone to gain consent for MR. If the parents gave verbal consent, a pre-paid envelope with consent form and information leaflet was sent to the parents (see reference 35 for full details of this process). Once MR consent was obtained, a post mortem MR was performed prior to standard autopsy. Great Ormond Street Hospital and Institute of Child Health Research Ethics Committee (04/Q0508/41) approved the study.

### Post mortem MR imaging

The first 20 subjects were used to optimise the imaging sequences. These subjects will not be included in the main study. The optimised MR study protocol is given in Table 1. A team of four specialist radiologists (paediatric neuroradiology, paediatric cardiology, paediatric chest & abdominal imaging and musculoskeletal imaging) Each reported the MR image, blinded to the autopsy report. Each radiologist reported the post mortem MR independently on to a large Microsoft access database (Microsoft Inc, Redmond, USA), with predefined drop down menus of categorical variables and codes (based on standard autopsy reporting). The MR data will be then re-classified jointly by a radiologist and a pathologist with regards to the likely final diagnosis, broad diagnostic categories and specific abnormalities of each organ system.

**Table 1:** Sequences for post mortem magnetic resonance imaging

Sequence	Voxel size	TA (min)	TR (ms)	TE (ms)	Flip angle^0^	Averages
BRAIN IMAGING						

3 D CISS	0.6 × 0.6 × .06 mm	13.5	9.2	4.6	70	4

3D Flash T_1_W	1 × 1 × 1 mm	5.4	11	4.9	15	3

2D Destir T_2_W	0.4 × 0.4 × 0.4 mm	13.5	5460	14,115	150	6

GE (Haem)	0.5 × 0.4 × 4 mm	6.3	800	26	20	4

DWI	B = 0, b = 500, b = 1000					

SPINE IMAGING						

2D T_2_-W TSE (children only)	1 × 1 × 3 mm	5.43	3050	109	170	3

3D CISS (fetus only)	0.6 × 0.6 × 1 mm	4.2	9.1	4.5	70	8

3D T_1_-W Flash	0.6 × 0.6 × 1 mm	3.5	11	5.3	15	10

BODY IMAGING						

T_2 _W TSE	0.8 × 0.8 × 0.8 mm	6.2	3500	276		2

3D CISS	0.8 × 0.8 × 0.8 mm	5.2	5.2	2.3	54	3

3D T_1_W VIBE	0.8 × 0.8 × 0.8 mm	5.5	5.9	2.4	25	8

3D CISS (cardiac)	0.6 × 0.6 × 0.6 mm	29	5.6	2.5	54	10

TA: Time for acquisition, TR: Relaxation time, TE: Echo time, ms: milli second, min: minutes

CISS: Constructive Interference Steady State, GE: Gradiant Echo, TSE: Turbo spin echo, DWI: Diffusion weighted imaging

### Conventional autopsy

Experienced paediatric or perinatal pathologists performed all autopsies according to the Royal College of Pathologists' guidelines, with input from specialist paediatric cardiac pathologists or neuropathologists as required. The pathology data were entered into the same database using an independent access portal, blinded to the MR data. The categorisation will be the same as for the MR imaging (i.e. specific final diagnosis, broad diagnosis category and separate system normal/abnormal rating) (Table 2). This provides the gold standard against which the MR imaging is assessed.

**Table 2:** Specific diagnostic categories

Code	Diagnosis
1	Normal

2	Abnormal

3	Non Diagnostic

4	Not confirmed

5	Not examined

6	Unexplained

7.01	Abdomen Abdominal wall defect

7.02	Abdomen Acute Intra abdominal pathology

7.03	Abdomen Adrenal haemorrhage

7.04	Abdomen Cloacal extrophy

7.05	Abdomen Dilated gut

7.06	Abdomen Duodenal atresia

7.07	Abdomen Exompholos

7.08	Abdomen Gastroenteritis

7.09	Abdomen Gastroschisis

7.1	Abdomen Gut infarction

7.11	Abdomen Hepatic necrosis

7.12	Abdomen Hepatomegaly

7.13	Abdomen Intestinal atresia

7.14	Abdomen Intestinal obstruction

7.15	Abdomen Large kidneys

7.16	Abdomen Liver artefacts

7.17	Abdomen Liver haemangioma

7.18	Abdomen Malrotation

7.19	Abdomen Meckel's Diverticulum

7.2	Abdomen Necrotising enterocolitis

7.21	Abdomen No spleen

7.22	Abdomen Peri-portal abnormality

7.23	Abdomen Rectal obstruction

7.24	Abdomen Small adrenals

7.25	Abdomen Splenomegaly

7.26	Abdomen Strangulated hernia

7.27	Abdomen Subcapsular haematoma

7.28	Abdomen Traumatic abdominal wall defect

7.29	Abdomen Volvulus/malrotation

7.3	Abdomen Other

8.01	Brain Acquired brain damage

8.02	Brain Aqueductal stenosis

8.03	Brain Bilateral thalamic damage/haemorrhage

8.04	Brain Brain malformation non specified

8.05	Brain Brain stem encephalitis

8.06	Brain Callosal agenesis

8.07	Brain Cap Haemangioma/Leukoencephalopathy

8.08	Brain Cerebellar abnormality

8.09	Brain Cerebellar fossa cyst

8.1	Brain Cerebellitis

8.11	Brain Cerebral infarction

8.12	Brain Chronic Brain Injury

8.13	Brain Complex neuropathological changes

8.14	Brain Congenital brain malformation

8.15	Brain Congenital brain malformation-Specific diagnosis

8.16	Brain Cortical maldevelopment

8.17	Brain Cranial vascular Malformation

8.18	Brain Dandy walker syndrome/variant

8.19	Brain Destructive brain lesion

8.2	Brain Diffuse brain injury

8.21	Brain Dural sinus malformation

8.22	Brain Globus pallidus abnormal

8.23	Brain Head Injury

8.24	Brain Infective brain lesion

8.25	Brain Inter-hemispheric Arachnoid cyst

8.26	Brain Intracranial bleed

8.27	Brain Ischemic brain injury

8.28	Brain Lissencephaly

8.29	Brain Microcephaly

8.3	Brain Microlissencephaly

8.31	Brain Neurological abnormality NOS

8.32	Brain Non-obstructive hydrocephalus

8.33	Brain Neural tube defect

8.34	Brain Old brain injury

8.35	Brain Polymicrogyria

8.36	Brain Pontine calcification

8.37	Brain Porencephaly

8.38	Brain Preterm brain injury

8.39	Brain Ruptured cerebral aneurysm

8.4	Brain Schizencephaly

8.41	Brain Schizencephaly/Septo optic dysplasia

8.42	Brain Subdural bleed

8.43	Brain Small cerebellum

8.44	Brain Spinal dysraphism

8.45	Brain Spinal intrathecal haemorrhage

8.46	Brain Tentorial tear

8.47	Brain Thalamic bleed

8.48	Brain Ventriculomegaly

8.49	Brain Vermis hypoplasia

8.5	Brain White matter lesions

8.51	Brain Other

9.01	Chest Aspiration

9.02	Chest Chronic lung disease

9.03	Chest Cytomegalovirus pneumonitis

9.04	Chest Congenital diaphragmatic hernia

9.05	Chest Congenital neck malformation

9.06	Chest Consolidation

9.07	Chest Cystic hygroma

9.08	Chest Drowning

9.09	Chest Hyaline membrane disease

9.1	Chest Lung lesion

9.11	Chest Meconium aspiration

9.12	Chest Pneumonia

9.13	Chest Pneumonia/Specific virus

9.14	Chest Pulmonary congestion and edema

9.15	Chest Pulmonary haemorrhage

9.16	Chest Pulmonary hypertension

9.17	Chest Small lungs/Hypoplasia

9.18	Chest Sub glottic stenosis

9.19	Chest Tracheo oesophageal fistula

10.01	Genetic Body stalk anomaly

10.02	Genetic Chromosomal abnormality

10.03	Genetic Other mutations

10.04	Genetic Genetic Syndrome

10.05	Genetic Other

10.06	Genetic Larson-like syndrome

10.07	Genetic Miller Dieker syndrome

10.08	Genetic Palister-Hall syndrome

10.09	Genetic Pallister-killian syndrome

10.1	Genetic Sirenomelia

10.11	Genetic TRAP

10.12	Genetic Trisomy 18

10.13	Genetic Trisomy 21

10.14	Genetic Turner's syndrome

11.01	Haematology Congenital leukaemia

11.02	Haematology Fetal anaemia/Parvo virus

11.03	Haematology Fetomaternal bleed

11.04	Haematology Haematological

11.05	Haematology Myelodysplastic syndrome

11.06	Haematology Rhesus isoimmunisation

12.01	Heart Aortic Valvular stenosis

12.02	Heart Cardiac ion channelopathy

12.03	Heart Atrial septal defect

12.04	Heart Atrioventricular septal defect

12.05	Heart Bi ventricular hypertrophy

12.06	Heart Blocked cardiac shunt

12.07	Heart Cardiac abnormality

12.08	Heart Cardiac Teratoma

12.09	Heart Cardiac Tumor

12.1	Heart Cardiomegaly

12.11	Heart Cardiomyopathy

12.12	Heart Coarctation

12.13	Heart Common arterial trunk

12.14	Heart Complex Congenital heart disease

12.15	Heart Congenital heart disease non specified

12.16	Heart Cortriatrum

12.17	Heart Dextrocardia

12.18	Heart Dilated cardiomyopathy

12.19	Heart Double outlet right ventricle

12.2	Heart Hypoplastic left heart syndrome

12.21	Heart Left atrial isomerism

12.22	Heart Myocardial infarction

12.23	Heart Myocarditis

12.24	Heart Narrowed cardiac shunt

12.25	Heart Pulmonary atresia

12.26	Heart Persistent left superior vena cava

12.27	Heart Restrictive foramen ovale

12.28	Heart Retro oesophageal right subclavian

12.29	Heart Right isomerism

12.3	Heart Partial anomalous venous drainage

12.31	Heart Situs inversus

12.32	Heart Situs inversus/Congenital heart disease

12.33	Heart Total anomalous venous drainage

12.34	Heart Tetrology of Fallot

12.35	Heart Transposition of great arteries

12.36	Heart Tricuspid Atresia

12.37	Heart Ventricular septal defect

12.38	Heart Ventricular septal defect/Coarctation

13.01	Metabolic specific metabolic diagnosis

13.02	Metabolic Steatosis/Non specific Metabolic diagnosis

14.01	Musculoskeletal Arthrogyposis

14.02	Musculoskeletal Cleft palate

14.03	Musculoskeletal Cleft vertebrae

14.04	Musculoskeletal Congenital myasthenia gravis

14.05	Musculoskeletal Fracture skull

14.06	Musculoskeletal Fracture long bones

14.07	Musculoskeletal Lacerated muscle

14.08	Musculoskeletal Other

14.09	Musculoskeletal Osteogenesis imperfecta

14.1	Musculoskeletal Osteogenesis imperfecta type II

14.11	Musculoskeletal Osteogenesis imperfecta type IIa

14.12	Musculoskeletal Osteogenesis imperfecta type IIb

14.13	Musculoskeletal Myopathy

14.14	Musculoskeletal Rib fractures

14.15	Musculoskeletal Shoulder dystocia

14.16	Musculoskeletal Skeletal dysplasia

14.17	Musculoskeletal Short limb dysplasia non specific

14.18	Musculoskeletal Talipes

14.19	Musculoskeletal Thanatophoric dysplasia

14.2	Musculoskeletal Thanatophoric dysplasia like osteochondrodysplasia

14.21	Musculoskeletal Thanatophoric dysplasia type 1

15.01	Other Hydrops

15.02	Other Ichthyosis

15.03	Other Intrauterine growth retardation

15.04	Other multi organ failure

15.05	Other Prematurity

15.06	Other Surgical emphysema

16.01	Placenta Other

16.02	Placenta Chorioamnionitis/Funisitis

16.03	Placenta Cord prolapse

16.04	Placenta Fetal thrombotic vasculopathy

16.05	Placenta Histiocytic intervillositis

16.06	Placenta Infarction

16.07	Placenta Placental abruption

16.08	Placenta Placental pathology-Infective

16.09	Placenta Placental pathology-Non Infective

16.1	Placenta Prolonged rupture of membranes

16.11	Placenta Spontaneous rupture of membranes

16.12	Placenta Utero placental disease

16.13	Placenta Villitis of Unknown aetiology

17.01	Renal Bladder outlet obstruction

17.02	Renal Congenital renal malformation

17.03	Renal Cystic kidney disease

17.04	Renal Focal renal dysplasia

17.05	Renal Focal renal non specified

17.06	Renal Large kidneys

17.07	Renal Obstructive uropathy

17.08	Renal Renal adysplasia

17.09	Renal Renal agenesis

17.1	Renal Renal developmental abnormality

17.11	Renal Renal dysplasia

17.12	Renal Renal tubular necrosis

17.13	Renal Syndromic Cystic Renal Dysplasia

18.01	Sepsis Cytomegalovirus

18.02	Sepsis Adeno virus infection

18.03	Sepsis Herpes simplex virus

18.04	Sepsis Intra uterine infection: Non specified

18.05	Sepsis Sepsis

18.06	Sepsis Sepsis/E coli

18.07	Sepsis Sepsis/Group B Streptococci

18.08	Sepsis Other Streptococcal infection

18.09	Sepsis Toxoplasmosis

18.1	Sepsis Cytomegalovirus

19.01	Trauma Hanging

19.02	Trauma Non accidental injury

19.03	Trauma Traumatic other

20.01	Tumor Sacro coccygeal teratoma

20.02	Tumor Other tumors

### Data analysis

Using conventional post mortem as the gold standard comparator, the MR images will be assessed for accuracy of the anatomical morphology, associated lesions, clinical usefulness of information and determination of the cause of death. The primary outcome will be the percentage of cases for which MR imaging correctly identifies the diagnostic category. Secondary analyses will make more detailed comparison of the individual measurements recorded within both autopsy types to facilitate understanding of how different diagnoses may occur. The sensitivities, specificities and predictive values of: (i) MR imaging plus clinical history, and (ii) MR imaging plus clinical history and other non-invasive post mortem investigations (e.g. external examination, genotyping, placental examination and skeletal survey) and to identify: (a) the specific diagnosis (b) one of predefined broad categories of diagnosis and (c) specific diagnostic category for each system, will be presented.

Changes according to weight of the fetus/infant/child will be investigated using logistic regression models. Diagnostic statistics will also be presented for the individual components (brain, chest, abdomen, heart and musculoskeletal) to determine areas of good and bad concordance. All estimates will be presented with 95% confidence intervals. Ante mortem assessments will be similarly compared with those made post mortem.

### Sample size calculations

To determine the primary outcome of percentages correctly diagnosed to within +/- 5% with 95% confidence will require 400 cases if the percentage correct is as low as 50%. We anticipate that the percentage correct will be substantially higher than this, in which case the estimate will be more precise. If the percentage correct is as high as 90%, then this will be estimated to within +/- 3% with a sample of 400.

Similarly for the individual components, we anticipate a wide range in the age distribution of cases and this should allow some quantification of changes by weight. Further investigations within specific diagnoses will necessarily be based on smaller numbers and are hence more exploratory than definitive in nature. Presentation with confidence intervals will indicate any under-powering of the comparisons.

## Discussion

Over the past decade, the consent rate for autopsy in the newborn has been less than 20% in the UK [8]; pediatric autopsies have become virtually non-existent, apart from the cases investigated by the HM Coroners or police (forensic cases), where parental consent is not required. Furthermore, this decline has occurred despite an increase in the number of cases in which autopsy consent (approximately 80% of cases following a perinatal death) is sought by the clinicians. One of the key reasons for parental refusal is the apparent invasive nature of conventional autopsy.

Although post mortem MR imaging was reported as an alternative for conventional autopsy more than a decade ago, it has not been introduced into routine clinical practice as supporting evidence is based on small and poorly designed studies [35]. In the UK, its use has been limited to some private initiatives. The Chief Medical Officer (UK) recommended rigorous evaluation of post mortem MR imaging as an alternative for autopsy, before it is widely introduced into the UK clinical practice. Following this, the UK Department of Health funded the present study (MaRIAS-Magnetic Resonance Imaging Autopsy Study) for systematic and rigorous evaluation of post mortem MR imaging as an alternative for conventional autopsy with a view to making recommendations to the Department of Health with regard to the advisability of introduction into routine clinical practice, either on its own or along with other minimally invasive post mortem investigations [37].

## List of Abbreviations

CESDI: Confidential Enquiry into Stillbirths and Deaths in Infancy; CISS: Constructive Interference Steady State; DWI: Diffusion weighted imaging; GE: Gradiant Echo; GOSH: Great Ormond Street Hospital for Children NHS Trust; HM Coroner: Her Majesty's Coroner; MR: Magnetic resonance; PROM: Prolonged rupture of membranes; RCOG: Royal College of Obstetricians and Gynaecologists; TA: Time for acquisition; TE: Echo time; TR: Relaxation time; TSE: Turbo spin echo; UCH: University College Hospital Foundation NHS Trust; UCL: University College London.

## Competing interests

The authors declare that they have no competing interests.

## Authors' contributions

AMT prepared the grant application along with LSC, NJR and NJS. AMT (Chief Investigator) has overall responsibility for the study and interpreted the cardiac images. ST conducted the study as a part of his PhD work under supervision of AMT and dealt with all aspects of the study including drafting of the study protocol, developing data collection systems, recruitment of the cases, MR imaging, collection of autopsy data and provided input into the clinical aspects of the study. ST will undertake the final data analysis under supervision of AW. ST and AMT will draft the final manuscript for publication and will be the guarantors of the study.

NJS provided input into the pathological aspects of the study and re-classified the MR data and pathology along with AMT. LSC assisted recruitment of fetal cases and provided input regarding fetal ultrasound. NJR assisted recruitment of neonatal cases and provided input into the neonatal aspects of the study. AW advised on the study design, statistical aspects and will oversee the final data analysis. RG, KC and DS reported the brain and spinal cord MRI; CO and OO interpreted chest and abdomen MRI and AO interpreted musculoskeletal MRI. All authors have contributed to the protocol development and have approved the final version of the study protocol submitted for publication.

## Quality assurance of the data

Transparent Research Audit System (TRUST) guidelines will be followed for analysis, authorships and publication of the MaRIAS data. AMT re-examined all MR images and cross checked against the radiology reports on all organ systems for quality assurance. Shea Addison (SA) will cross check all the pathology data for any data entry errors. AMT, NJS and SA will be responsible for cleaning the data. The final data used for analysis and accurate contributions of each author will be stored at UCL until 2032 for research data auditing. ST and AMT will be the custodians of the MaRIAS data. All manuscripts from the study will be reviewed by the Department of Health (UK), ST and AMT before submission, though the Department of Health (UK) does not influence the scientific content of any research output.

## Appendix 1: Information leaflet

VERSION 1.0

28.4.2007

xxxxxxxx

Post mortem Magnetic Resonance Imaging in fetuses, newborn and Children: A comparative study with conventional autopsy.

Information leaflet for parents

Thank you for taking the time to read this leaflet. We know that this is a difficult time for you and appreciate the time you are taking to read this leaflet.

*Background to the study*

MRI (Magnetic resonance imaging) and CT scans, as you may be aware are special techniques to get images of the body. An MRI scan can examine internal organs in detail and may be able to identify some of the problems that can be detected by a post mortem examination. In some cases, we believe that MRI may even be better than a post mortem examination. Many parents are understandably upset about the thought of their baby undergoing a post mortem. We are doing this study to find out if an MRI scan of the whole body can give similar information to that of post mortem, so that in future we might be able to offer an MRI scan instead of post mortem.

*What will happen if we agree to take part?*

If you agree to take part we will arrange for your baby to have an MRI scan (and in some cases a CT scan as well) as soon as possible at Great Ormond Street Hospital for Children. This involves taking a series of pictures using a special machine. We may take biopsy using small needles under MRI guidance, for examination under microscope. The whole process will take about 2 hours. As soon as the scan is done we will arrange for your baby to be taken for the traditional post mortem. We will ensure that at all times your baby will be treated with due respect and reverence.

The MRI scan will not delay the post mortem or the timing of burial or cremation. Taking part would not involve you in any extra hospital visits. Any additional information, if any from MRI/CT scan will be included in autopsy report to the coroner. We will also need to have access to the post mortem results and the results of any other tests that were done before or after birth. This is so that we can compare the results of tests which are done traditionally with the results from the MRI and work out which combination of tests give the most accurate results overall.

*Will my taking part in this study be kept confidential?*

All information that is collected about you or you baby or during the course of the research will be kept strictly confidential. Any information we collect will only be used by the research team for the purpose of the study.

Who will have access to the case/research records?

All the data and images collected as part of this study will be stored on a secure computer. Only the researchers involved in this study will have access to the data collected in the course of this study. A representative of the hospital's Research Ethics Committee will also have access to data. The 1988 Data Protection Act safeguards the use of some types of personal information. This places an obligation on those who record or use personal information, but also gives rights to people about whom information is held. If you have any questions about data protection, please contact the Data Protection officer via the switchboard on 0845 155 5000. The results from our project will be published as papers in medical journals. No data will be published that allows for individuals to be identified in any way. If requested, we will be able to send you copies of any papers published when we have completed the study in 3-4 years time.

Do you have to take part?

It is up to you to decide whether or not to take part. If you do decide to take part you will be given this information sheet to keep and will be asked to sign a consent form. You will be given a copy of the signed consent form for your records. If you do not feel able to take part it will not in any way affect the care your family receives.

Who do I speak to if I have further questions or worries?

In the first instance please contact xxx who is coordinating this project. His contact details are given below. xxx can also be contacted if you need any further information or xxx is not available. If you wish to speak to someone not directly involved in the study then please contact xxxxx

If you have any complaints about the way in which the project is being or has been conducted, in the first instance please discuss them with any of the doctors listed below. If the problems are not resolved, or you wish to comment in any other way please contact xxxx

*Who is organising and funding the research?*

This study is being organised by the Cardiothoracic, Radiology and Pathology Departments at Great Ormond Street Hospital for Children and by the Fetal and Neonatal Medicine Units and Pathology Department at University College London Hospital, Funding is provided by the Department of Health. This study has been reviewed and approved by the Great Ormond Street Hospital Research Ethics Committee.

Thank you once again for all your time and trouble.

Contacts for further information: XXXX

## Appendix 2: Consent form

Version 1.0

REC reference number: **04/Q0508/41**

Study R & D Number: **04CC20**

Patient Name....................

Unit Number....................

Date of Birth....................

Patient Identification Number for this trial:.................

*CONSENT FORM*

Title of Project: Post mortem magnetic resonance imaging in the fetus, infant and child: A comparative study with conventional autopsy

1. I confirm that I have read and understand the information sheet dated 28/9/07 (version 1.0) for the above study, and have had the opportunity to ask any questions.    ☐

2. I understand that my participation is voluntary, and that I am free to withdraw at any time, without giving any reason, without my medical care or legal rights being affected.    ☐

3. I understand that sections of baby's medical notes may be looked at by responsible individuals named in the study or by from regulatory authorities from the Trusts. I give permission for these individuals to have access to baby's records.    ☐

4. I agree to take part in the above study.

________________________   ____   ________________

Name of Parent/Legal Guardian     Date     Signature

______________________________________________   ____   _____________

Name of Person taking consent (if different from Researcher)     Date     Signature

_________   ____   ________________

Researcher     Date     Signature

1 for Patient; 1 for Researcher; 1 to be kept with Hospital Notes

## Pre-publication history

The pre-publication history for this paper can be accessed here:

http://www.biomedcentral.com/1471-2431/11/120/prepub
